# Sarcoma Happens: A Reminder for Arthroscopic Surgeons

**DOI:** 10.7759/cureus.24457

**Published:** 2022-04-25

**Authors:** Christa L LiBrizzi, Alexander M Bitzer, R. Timothy Kreulen, Christian F Meyer, Carol D Morris

**Affiliations:** 1 Orthopaedic Surgery, Johns Hopkins University, Baltimore, USA; 2 Orthopaedic Surgery, West Virginia University, Martinsburg, USA; 3 Oncology, Johns Hopkins University, Baltimore, USA

**Keywords:** tumor, sarcoma, knee, intra-articular, arthroscopy

## Abstract

Primary intra-articular sarcomas are rare and present with nonspecific symptoms such as pain or swelling. Due to nonspecific symptoms, patients may undergo routine diagnostic arthroscopy, which ultimately leads to sarcoma diagnosis. Here we present four patients with intra-articular sarcomas of the knee diagnosed after arthroscopy. The goal of this study is to highlight the importance of including malignant bone and soft-tissue sarcomas in the differential diagnosis of patients with nonspecific knee symptoms.

A case series was developed from a retrospective review of prospectively collected data from our institution’s orthopedic oncology database. Patients who underwent arthroscopic procedures on the knee and who were diagnosed with intra-articular sarcomas postoperatively from 2014 to 2019 were identified. All patients underwent diagnosis, staging, and multidisciplinary evaluation and treatment. Clinical characteristics, oncologic considerations, and surgical outcomes are described.

Four patients with intra-articular sarcomas of the knee diagnosed after arthroscopy for non-oncologic concerns were identified: two synovial sarcomas, one Ewing sarcoma of bone, and one osteosarcoma. All surgical plans and treatment options were significantly affected by the previous arthroscopic procedures. One patient underwent above-the-knee amputation; one patient underwent extra-articular wide resection of the knee, including portal sites with distal femur/total knee reconstruction; one patient underwent rotationplasty, and one patient was treated with therapeutic radiation (no surgery). All patients received chemotherapy.

Although intra-articular sarcomas are rare, orthopaedic surgeons must remain vigilant when proceeding with arthroscopic procedures if the clinical history, physical exam, and imaging findings are not perfectly aligned.

## Introduction

Primary intra-articular masses are rare and most often occur in the knee [1]. Lesions commonly found in the knee are largely benign and include pigmented villonodular synovitis (PVNS) and synovial chondromatosis [2,3]. However, intra-articular primary sarcomas of the knee can also occur, including synovial sarcoma [4], osteosarcoma [5], extraskeletal chondrosarcoma [6], and epithelioid sarcoma [7]. Often these malignant masses are difficult to distinguish from benign tumors due to considerable overlap in clinical presentation [4,6,8].

Patients with intra-articular knee masses typically present with nonspecific pain, swelling, and/or a sense of instability [9]. They may present months to years after an insidious onset or after an acute change in knee function as a result of sporting activities or an acute injury [10,11]. Radiographs obtained at evaluation may show subtle soft-tissue shadowing, calcifications, or displacement of normal surrounding structures because of mass effect, but radiographs often appear normal [2]. Advanced imaging, such as magnetic resonance imaging (MRI), can also appear benign and nonspecific [12]. Thus, patients may choose to undergo procedures such as intra-articular injections or diagnostic arthroscopy with subsequent debridement [13]. In the presence of an intra-articular sarcoma, these elective procedures risk contamination of normal uninvolved tissue with tumor cells. This scenario may later require a more morbid surgery than would have been necessary if invasive treatment had been deferred before diagnosis [11,14]. Therefore, early recognition of an intra-articular sarcoma is essential to prevent future morbidity and maximize functional and oncological outcomes.

We describe four patients who underwent arthroscopic procedures on the knee who were diagnosed postoperatively as having intra-articular sarcomas. We aim to report on primary intra-articular sarcomas specific to the knee and to highlight the importance of including malignant bone sarcomas and soft-tissue sarcomas (STS) in the differential diagnosis for patients with nonspecific knee symptoms and ambiguous imaging.

## Case presentation

Using our institution’s orthopedic oncology database, we performed a retrospective review of prospectively collected data from 2014 to 2019. We included patients with primary bone sarcoma or STS with intra-articular knee involvement who underwent knee arthroscopy before obtaining an oncologic diagnosis. All patients underwent diagnosis, staging, and multidisciplinary evaluation and treatment (Table 1). Clinical characteristics, oncologic considerations, and surgical outcomes are described.

**Table 1:** Characteristics of four patients with primary intra-articular sarcomas of the knee ACL: anterior cruciate ligament; AWD: alive with disease; BPB: bone-patella-bone; NED: no evidence of disease.

Patient	Initial Diagnosis	Arthroscopic Procedure	Final Diagnosis	Adjuvant Treatment	Surgical Management	Metastasis	Time Disease Free, Status	Final Surgical Management Affected
1	Acute meniscus tear, cartilage defect with possible ganglion or parameniscal cyst	Diagnostic arthroscopy with partial mass excision	Synovial sarcoma	Chemotherapy	Extra-articular wide resection and reconstruction	No	72 months, NED	Yes
2	Pigmented villonodular synovitis	Knee arthroscopy with partial resection	Synovial sarcoma	Chemotherapy	Above-knee amputation	No	42 months, NED	Yes
3	ACL injury	ACL reconstruction, BPB autograft, partial meniscectomy, arthroscopic-assisted microfracture	Ewing sarcoma	Chemotherapy and radiation therapy	None	Yes, spine	24 months, AWD	Yes
4	ACL injury	ACL reconstruction, BPB autograft	Osteosarcoma	Chemotherapy	Rotationplasty, converted to AKA	No	20 months, NED	Yes

Patient one

**Figure 1:**
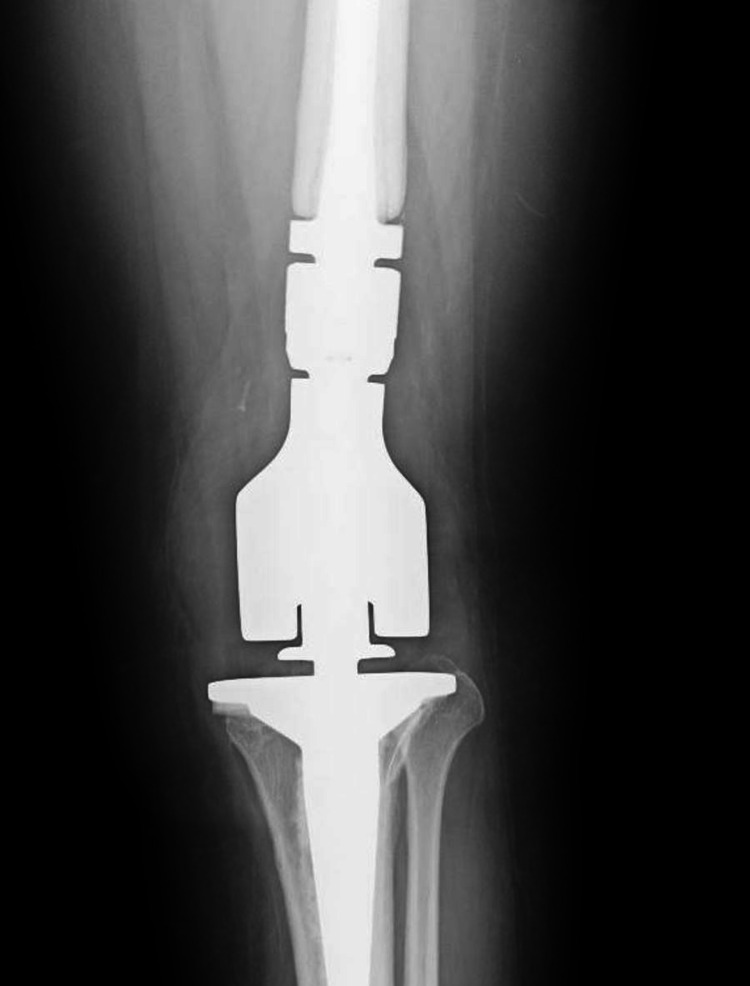
Anteroposterior radiograph of a 30-year-old woman (patient one) who underwent revision surgery, including wide excision and limb reconstruction after arthroscopic partial excision and debridement of a primary synovial sarcoma.

A 30-year-old woman presented for evaluation of knee pain and swelling lasting three months without antecedent trauma. During the initial consultation, radiographs of the knee were obtained and illustrated no acute abnormality. She was counseled on nonoperative management and advised to follow up. At follow-up, the knee had not improved with nonoperative treatment, and an MRI was obtained. The working diagnosis at the time was a meniscus tear or a cartilage defect. MRI of the left knee showed a 1.4×1.4×0.7-cm iobulated mass with heterogeneous signal intensity and deep to the retinaculum. The radiologist’s interpretation was a ganglion or parameniscal cyst with otherwise normal-appearing intra-articular structures. The patient underwent diagnostic arthroscopy with partial mass excision. Postoperatively, pathology revealed high-grade synovial sarcoma. The patient was subsequently referred for staging studies which were negative for the disease. She underwent preoperative chemotherapy and subsequent extra-articular wide resection, including the portal sites, and reconstruction with a distal femur replacement (Figure 1). The patient remains without evidence of disease at 72 months, requiring a hinged knee brace for ambulation secondary to instability.

Patient two

**Figure 2:**
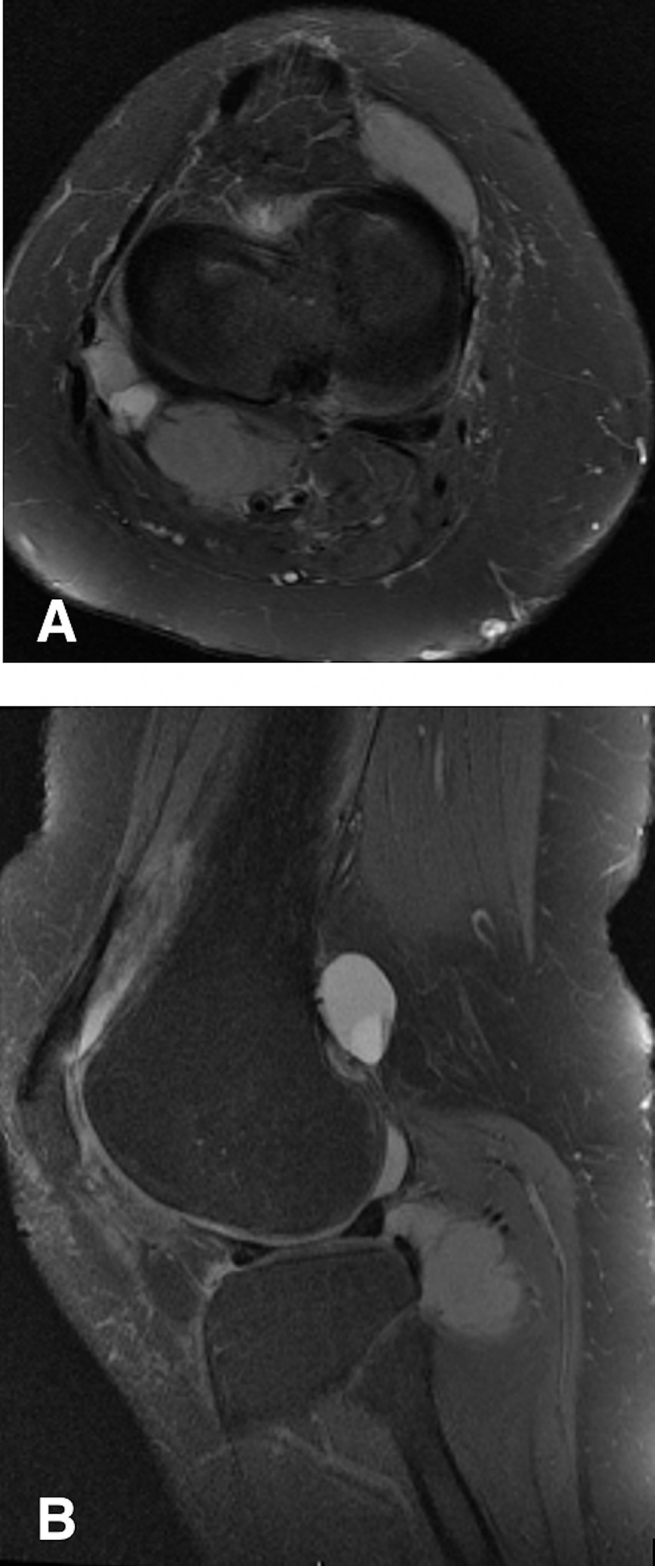
(a) T2-weighted, fat-suppressed axial, (b) proton density, fat-suppressed sagittal magnetic resonance images of the right knee in a 47-year-old woman (patient two). Images show multiple residual tumor lesions after previous arthroscopic debridement.

A 47-year-old woman presented to her primary care physician for generalized pain in her knee for months. Ultrasonography of the knee showed an intra-articular mass and a complex effusion. An MRI was obtained where multiple lobulated masses were found throughout the knee joint and were thought to be PVNS. The patient underwent knee arthroscopy with partial resection of multiple masses throughout the synovium and chondroplasty. Postoperatively, pathology was consistent with synovial sarcoma. MRI was obtained to determine the remaining tumor burden (Figure 2). Staging studies were negative for other sites of disease. The patient underwent preoperative chemotherapy and later above-knee-amputation (AKA). Pathology of the gross specimen after AKA showed multiple irregular tan-white masses within the knee joint. The patient currently ambulates independently with a prosthesis and remains without evidence of disease at 42 months.

Patient three

A 29-year-old man presented to our emergency department with severe back pain, bilateral leg radiculopathy, and bladder and bowel incontinence secondary to a large epidural mass at T11-L1. Biopsy obtained at the time of spinal canal decompression showed Ewing sarcoma. Further investigation uncovered a five-year history of right knee injury and pain, for which he underwent anterior cruciate ligament (ACL) reconstruction with ipsilateral bone-patella-bone autograft and partial meniscectomy. The pain did not resolve. He was subsequently treated with arthroscopic-assisted microfracture of the medial femoral condyle for presumed avascular necrosis.

**Figure 3:**
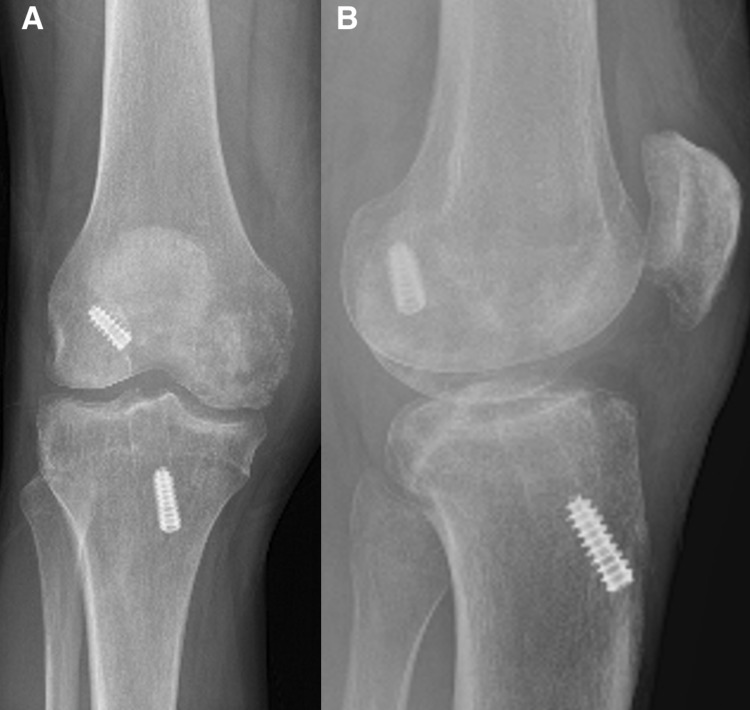
(a) Anteroposterior and (b) lateral radiographs of the right knee of a 29-year-old man (patient three) with previous anterior cruciate ligament reconstruction and a medial femoral condyle lesion.

**Figure 4:**
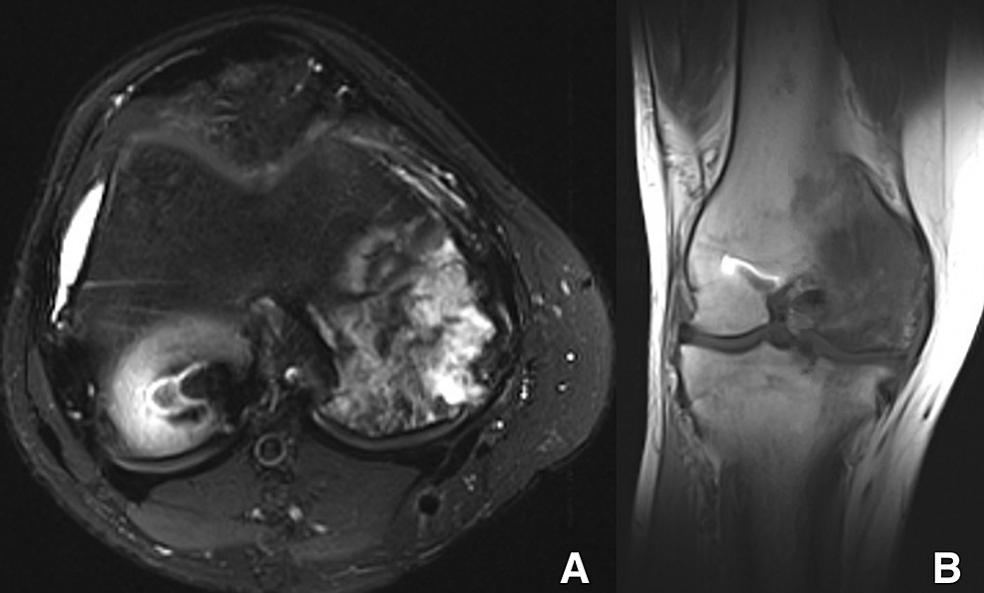
(a) T2-weighted fat-suppressed axial and (b) T1-weighted coronal magnetic resonance images of the right knee of a 29-year-old man (patient three) showing heterogeneity and hyperintensity on T2-weighted signal within the medial femoral condyle.

Radiographs of the right knee showed evidence of ACL reconstruction with retained instrumentation and a permeative lesion in the medial femoral condyle (Figure 3). MRI showed an enhancing infiltrative tumor within the distal medial femoral condyle with extension into the femoral notch and prominent perilesional edema consistent, with a primary bone sarcoma thought to represent the primary site of disease (Figure 4). The ACL graft was intact. The patient underwent an extended chemotherapy course and radiation to the spine, eliminating disease at all sites except the knee. Various local control options for the knee were discussed, including amputation, extra-articular knee resection and reconstruction, rotationplasty, and radiation only. He chose to undergo therapeutic radiation. He lived with the disease for more than 24 months after diagnosis and 13 months after the radiation therapy to his right femoral condyle.

Patient four

**Figure 5:**
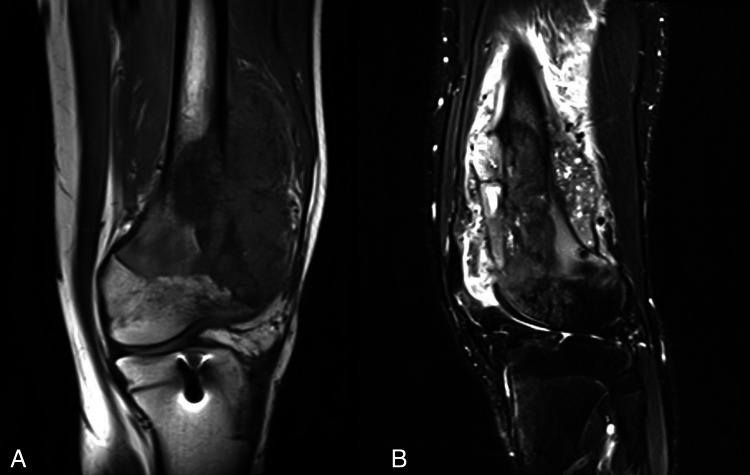
(a) T1-weighted coronal and (b) T2-weighted fat-suppressed sagittal magnetic resonance images of the left knee of a 16-year-old boy (patient four). Images show a T1-weighted hypointense and T2-weighted heterogenous lobulated mass involving the distal femoral metaphysis and lateral condyle after anterior cruciate ligament reconstruction.

A 16-year-old boy was referred to our institution for evaluation of a left knee mass. He had undergone ACL reconstruction with ipsilateral bone-patella-bone autograft 10 months earlier. Radiographs were obtained because of persistent pain and showed an irregular calcific density along the anterolateral diametaphysis of the distal femur with cortical disruption, periosteal elevation, and involvement of the surrounding soft tissue. There was no evidence of disease elsewhere throughout the femur. MRI showed a hyperintense T1/T2 lobulated osseous mass centered at the distal femoral metaphysis measuring 8.3 × 6.1 × 15.5 cm with intra-articular extension (Figure 5). A biopsy of the lesion confirmed high-grade osteosarcoma. Staging studies were negative for metastatic disease and skip lesions. He was started on preoperative chemotherapy. After an extensive discussion regarding local control options, he chose to undergo rotationplasty with later conversion to AKA. He remains without evidence of disease at 20 months post-surgery.

## Discussion

Bone sarcoma and STS account for less than one percent of all cancer diagnoses in adults, with the lower extremity being the most common site [15]. Sarcomas rarely occur as primary intra-articular masses but rather invade locally by direct extension along with intra-articular ligaments, direct articular extension along with the capsule, and hematoma formation [16,17]. The clinical presentation of intra-articular malignancies overlaps with more common sports-related injuries [10]. We aimed to report and characterize the clinical course of four patients who underwent arthroscopic procedures and were subsequently diagnosed with primary sarcomas of the knee with the hopes of reminding sports medicine surgeons to remain vigilant when the history, exam, and imaging findings are not perfectly aligned. The sarcomas in this series comprised of two soft tissue sarcomas, both of which were synovial sarcoma, and two bone sarcomas consisting of one osteosarcoma and one Ewing sarcoma.

The diagnosis of extra-articular or intra-articular neoplasms via arthroscopy has been previously described [7,10,13]. Lewis and Reilly [10] reported 36 patients with musculoskeletal symptoms attributed to sports injuries later diagnosed with primary neoplasms. Half of the lesions were malignant, the lower extremity was involved more than 90% of the time, and most patients (n = 22) had knee involvement. Diagnostic arthroscopy or arthrography was performed before tissue diagnosis in 70% of patients with malignant lesions around the knee. The authors noted a major delay in diagnosis due to the confounding history of injury. Joyce and Mankin [18] described 11 patients with undiagnosed extra-articular neoplasms who underwent knee arthroscopy. Neoplasms were malignant in seven patients. A retrospective review of radiographs found seven patients with bone lesions of soft-tissue masses. Of the seven imaging reports, only two mentioned these findings. Four patients were documented as having undergone arthroscopic intra-articular bone biopsies, although two patients required re-biopsy for inadequate sampling. No patients had documented metastasis into or across the joint, which is a theoretical risk with trans-synovial biopsy through an arthroscope. This series emphasizes the importance of obtaining adequate imaging and performing an appropriate evaluation of the imaging before surgery. In general, the clinical history and exam should match the suspected pathology. In cases where this is discordant, a high index of suspicion should lead to further workup. MRI is the most useful tool for identifying and characterizing bone and soft tissue tumors. Current MRI protocols incorporate several functional sequences to distinguish between malignant and benign conditions [2,19]. When indeterminate lesions are encountered, consultation with a musculoskeletal radiologist can guide subsequent appropriate imaging. Although intentional arthroscopic biopsy of a malignant lesion is contraindicated, obtaining diagnostic tissue after an intra-articular mass is encountered is mandatory.

The unintended violation of a compartment during invasive procedures and disruption of a malignant tumor may prevent a future limb-salvage procedure due to contamination of uninvolved tissue with tumor cells. Muscolo et al. [11] described 25 patients diagnosed with sports injuries who were treated with an intra-articular procedure and later found to have a knee mass. Fourteen patients had primary sarcomas around the knee, and nine of those patients progressed to a higher stage of disease after the index surgery before an evaluation by an orthopedic oncologist. The authors determined the arthroscopic procedure affected the final surgical plan in 27% of patients and precluded limb salvage. In our series, all surgical plans and treatment options were affected by previous arthroscopic procedures. Patient 1 would have had a less complex soft-tissue and extra-articular knee resection, Patient 2 would have undergone an extra-articular knee resection, and Patients 3 and 4 would have undergone intra-articular resections with reconstructions if their sarcomas were diagnosed before arthroscopic procedures. The more extensive the arthroscopic surgery, the larger the field of local contamination.

Treatment of intra-articular sarcomas can be achieved by extra-articular resection with endoprosthesis placement, rotationplasty, or amputation. Complete resection of the joint and contaminated soft tissue is necessary. Reconstruction after extra-articular resection is technically challenging and associated with high complication rates. Nottriott et al. [20] described limb salvage in eight patients with intra-articular STS of the knee. Five of these patients ultimately underwent amputation for infection control. Although extra-articular resection for intra-articular malignancies will likely be indicated for local control in the absence of arthroscopy, the additional soft-tissue contamination associated with arthroscopy adds a layer of complexity, especially as it pertains to soft tissue coverage and associated postoperative morbidity.

While our report is not novel, it serves as a contemporary reminder that in the current era, arthroscopic manipulation of sarcomas persists. We emphasize the importance of vigilance when clinical and radiographic presentations are discordant.

## Conclusions

Primary intra-articular sarcomas are rare and require a high level of suspicion from physicians. A thorough preoperative examination involving a patient history and physical examination, radiographs, and MRI should be obtained before arthroscopic instrumentation is performed in patients with nonspecific knee symptoms. If intra-articular malignancy is suspected, arthroscopic intervention should be avoided, and the patient should be referred for oncologic work-up and treatment. If a mass suspicious for malignancy is encountered unexpectedly during a surgical procedure, it is essential to obtain a biopsy and minimize the area of contamination.
